# An analysis of US net cancer drug launch prices and clinical efficacy and certainty of evidence from 2008 to 2022

**DOI:** 10.1093/haschl/qxaf051

**Published:** 2025-03-13

**Authors:** Sumaya Abuloha, Benjamin P Harvey, Shu Niu, Alaa Alshehri, Melissa Miri, Katherine Clifford, James Chambers, Mikael Svensson

**Affiliations:** Department of Pharmaceutical Outcomes and Policy, College of Pharmacy, University of Florida, Gainesville, Fl 32611, United States; Department of Clinical Pharmacy and Pharmacy Practice, College of Pharmacy, Yarmouk University, Irbid, 21163, Jordan; Institute of Health and Care Sciences, Sahlgrenska Academy, University of Gothenburg, Gothenburg, 405 30, Sweden; Department of Pharmaceutical Outcomes and Policy, College of Pharmacy, University of Florida, Gainesville, Fl 32611, United States; Department of Pharmaceutical Outcomes and Policy, College of Pharmacy, University of Florida, Gainesville, Fl 32611, United States; Department of Pharmacy Practice, College of Clinical Pharmacy, Imam Abdulrahman Bin Faisal University, Dammam, 31451, KSA; Department of Pharmaceutical Outcomes and Policy, College of Pharmacy, University of Florida, Gainesville, Fl 32611, United States; Center for the Evaluation of Value and Risk in Health, Tufts Medical Center, Boston, MA 02111, United States; Center for the Evaluation of Value and Risk in Health, Tufts Medical Center, Boston, MA 02111, United States; Department of Pharmaceutical Outcomes and Policy, College of Pharmacy, University of Florida, Gainesville, Fl 32611, United States; School of Public Health and Community Medicine, Institute of Medicine, University of Gothenburg, Gothenburg, 405 30, Sweden

**Keywords:** cancer drugs, uncertainty of evidence, drug prices, oncology, efficacy, value-based

## Abstract

Over the last 15 years, cancer drug prices have increased substantially in the United States (US), with question marks on whether this can be justified based on improved patient outcomes. This study aimed to analyze the relationship between US cancer drug net launch prices and clinical efficacy and certainty of evidence. FDA-approved cancer drug indications from 2008 to 2022 were extracted and matched with net launch price data, 5 distinct measures of clinical efficacy, and measures on the certainty of evidence around the clinical efficacy. Descriptive statistics and linear regression models were used to assess if higher net launch prices were associated with better patient outcomes. Cancer drug launch prices (net per 1 year/course of treatment) have increased from around $100 000 in 2008 to $200 000 in 2022. The results did not support the idea that drugs with higher net launch prices had more impressive clinical efficacy or more robust evidence around the clinical efficacy. US cancer launch prices do not seem to be value-based, which may imply distorted incentives for the allocation of research and development investments.

## Introduction

Prescription drug spending per capita in the United States (US) was around 1218 USD in 2022, around 60% higher than the average spending in other high-income countries such as Canada, the United Kingdom, France, and Germany. The higher spending in the US is primarily driven by higher prescription drug prices rather than differences in utilization.^1-4^ These substantially higher US drug prices drive up health insurance premiums and out-of-pocket (OOP) costs, and managing OOP drug costs is a primary cause of concern for many US citizens.^5^ Prescription drugs with particularly high OOP costs can also substantially increase patients’ risk of medical bankruptcies.^6^ This risk is particularly relevant for cancer drugs, for which prescription drug costs have also increased substantially over time, with a relatively higher price growth rate than drugs for most other conditions.^4,7,8^

From a value-based healthcare perspective, higher drug prices over time can be justified if drugs provide increasingly better patient outcomes, such as improved overall survival (OS) or health-related quality of life,^9^ or if the efficacy data is more robust with a stronger evidentiary basis.^10,11^ Value assessment frameworks often rely on a combination of the expected clinical benefit and the certainty of evidence, such as the value assessment framework used by the US Institute for Clinical and Economic Review^12^ and frameworks used by several European Health Technology Assessment agencies.^13,14^ Paying more for more effective drugs can also be important to incentivize efficient allocation of research and development (R&D) investments.^15^

Some evidence suggests that increasing drug prices over time is associated with improved drug efficacy. One study on anti-cholesterol drugs showed that increases in price from 1996 to 2007 were more than accounted for by improved clinical benefits.^16^ The relationship between price and clinical benefits has been more contested for cancer drugs. In a study trying to construct quality-adjusted price indexes, results indicated that increasing drug costs over time was motivated by accompanying efficacy improvements in colorectal cancer and second-line multiple myeloma but not for first-line multiple myeloma treatments.^17^ Another study examining 58 cancer drugs launched between 1995 and 2013 indicated that, on average, drugs with higher survival benefits were launched at higher prices^15^ and a study focusing on drug treatments for the 30 most prevalent cancers also documented a statistically significant positive association between efficacy and drug prices.^18^

However, more recently, several papers have assessed the correlation between launch and post-launch cancer drug prices in the US and various measures of clinical efficacy and where no, or at best, a weak relationship between increasing cancer drug prices and clinical benefits and certainty of evidence has been found. For example, studies correlating cancer drug prices and clinical benefit data for FDA-approved drugs between 2000 and 2015,^19,20^ 2015 to 2020,^21^ 2009 and 2013,^22^ 2006 and 2015,^23^ 2009 and 2017,^24^ and 2005 to 2023,^25^ did not find consistent statistically significant relationships between clinical benefits and prices. One study analyzing data from 2003 to 2022 found a (weak) correlation between clinical benefits and prices for original cancer drug indications but not when including subsequent indications,^26^ whereas another study documented a negative statistically significant association between efficacy assessments and prices.^27^ The certainty of evidence of the clinical benefit can be proxied by the study design of the pivotal trials and whether the clinical benefit has been demonstrated on a clinical endpoint. Several studies have documented a negative association between cancer drug prices and certainty of evidence, ie, lower prices for drugs with pivotal trial evidence from randomized study designs with data on clinical endpoints.^21,22,26^

This study aimed to add further evidence to whether cancer drug prices in the US are value-based, defining value in terms of the magnitude of clinical benefits and the certainty of evidence. This study makes two main contributions. First, as there is no “perfect” metric for assessing clinical benefits and previous studies have often relied on a single or closely related efficacy metrics, this study uses five different clinical benefit metrics that capture a broader range of perspectives of clinical benefits and robustness of evidence. While some of the efficacy metrics we use, their combined use facilitates more robust findings on the relationship between clinical efficacy and launch prices. Second, by analyzing how the clinical efficacy affects launch prices using data that approximates net prices (accounting for rebates and discounts), it is possible to remove the potential bias introduced by analyzing list prices, which has been the approach used in the previously published studies.

## Study data and methods

### Study sample

All new cancer-drug indications approved by the FDA from 2008 to 2022 were identified while excluding any drug that treats a toxicity or side-effect of cancer treatment, any bioequivalent for a new formulation, and any generic/biosimilar drug. This set of cancer-drug indications was then merged with net price data from the SSR Health database (see Launch price data)^28^ to form the study analysis sample.

### Launch price data

The launch prices were retrieved from the SSR Health net price database, approximating net prices for drugs supplied by publicly traded producers, thus excluding the minority of privately owned producers.^29^ Due to the different availability dates between the FDA approval and price data, we define the launch price as the first price data point in any of the 2 quarters following the FDA approval (expressed in 2023 US dollars based on the US Consumer Price Index for all items).

The launch price variable captures the cost per year or course of treatment based on the net price per unit and assuming the maximum annual/course dosage. The price is calculated for cycles representing a full year for all products where the label does not explicitly state a shorter treatment duration, for which the price is based on assuming the full treatment duration. When data on the net price was missing for the launch quarters but available for one or several other quarters in the database, we used the gross price for the launch quarter and assumed the same discount as for the closest subsequent quarter with available net price data. See the supplement for full details on the coding of the launch price variable (Online Note S1).

### Efficacy and certainty of evidence metrics

We screened the FDA Hematology/Oncology (Cancer) Approvals and Safety Notifications from 2008 to 2022 and extracted data on clinical efficacy and certainty of evidence based on a pre-structured data extraction template (Online Table S1) together with information on the drug name, date of approval, indication, name of pivotal clinical trials, study design type, and type of approval (accelerated vs regular).^30^ If the data of interest was unavailable on the notifications form, we examined the Drugs@FDA database to extract the data.

We used 5 different metrics to measure the drug efficacy: (1) OS, (2) progression-free survival (PFS), (3) overall response rate (ORR), (4) European Society for Medical Oncology Magnitude of Clinical Benefit Scale (ESMO-MCBS), and (5) Quality-adjusted life-year (QALY) gains. Given data availability, we collected as many of the 5 outcome metrics as possible for each drug.

For OS and PFS, we extracted the hazard ratio (HR) as the magnitude of clinical efficacy. We dichotomized the HR data to categorize drugs in the upper/lower half of the magnitude of clinical efficacy. For data on OS, an HR below 0.7 placed a drug in the upper half, and for data on PFS, an HR below 0.56 placed a drug in the upper half in terms of magnitude of clinical efficacy. For the ORR, we considered the percentage of patients in the intervention group with partial or complete response as the magnitude of clinical efficacy (continuous measure). The OS, PFS, and ORR data were all sourced from the public FDA database.^30^ If a drug was approved based on multiple pivotal trials, we used data from the trial with the most favorable results for the intervention drug. We extracted data on OS, PFS, and ORR irrespective if it was the primary or secondary outcome in the pivotal trials. We sourced the ESMO-MCBS scores from the public database, and this score is a grading system for cancer drugs that aim to distinguish drugs with substantial clinical benefits. The ESMO-MCBS weighs clinical trial data on OS, PFS, response rates, QoL, toxicities, etc. (depending on indication and line of treatment), where drugs with grades 4 and 5 for non-curative drugs and grades A and B for curative drugs were classified as cancer drugs with substantial clinical benefits.^31^ The QALY-gain data is based on a review of the published literature extracting information on incremental QALYs (life-years gained in full health) for the relevant indication compared with the standard of care at the time of drug approval. The full details of the review process have been described in previous work.^32^

Certainty of evidence reflects the strength and the trustworthiness of efficacy data. We extracted information on whether the FDA approval was based on a randomized controlled trial (RCT) as opposed to a single-arm trial. The assumption was that drugs approved based on an RCT, all else equal, provide stronger evidence that the drug causally improves outcomes.^33^ For sensitivity analyses, we relied on an alternate metric of certainty of evidence that measured whether the approval was based on a trial that showed clinical benefits in OS as opposed to a surrogate endpoint (such as PFS, ORR, etc.). The assumption is that drugs approved solely based on surrogate endpoint data have, all else equal, a lower certainty of evidence considering that surrogates used in many cancer drug-indications have not been validated for whether the drug improves patient-relevant outcomes such as OS or quality of life for the relevant indication.^34^

### Statistical analysis

We analyzed the time trends for the 5 efficacy metrics, the metrics of certainty of evidence, and the launch price to assess any relevant variations over time. We also plotted the launch price to the efficacy and certainty of evidence metrics to graphically show the relationships and calculated the *P*-values to test linear correlations. In addition, we specified a number of linear regression models to analyze the relationships between the log of the launch price, efficacy, and certainty of evidence. In the regression models, the launch price was logged to account for the skewness of the price variable. A set of additional covariates is also included in the regression models: orphan disease status, accelerated approval status, and the year of launch. Orphan disease status is a binary dummy variable reflecting if the drug indication received FDA orphan disease status and is sourced from the Drugs@FDA database.^30^ Accelerated approval is also a binary dummy variable capturing if the drug-indication received accelerated approval status, a “fast-track” regulatory pathway for drugs targeting an indication with unmet need and where the FDA does not require data on clinical endpoints.^35^ It should be mentioned that the FDA also increasingly has accepted surrogate endpoint data in the regular approval pathway.^36^ The launch year variable can capture time trends and show average year-to-year price changes in launch prices.

In sensitivity analyses, we separated first and supplemental indications and assessed the robustness using a broader set of generalized linear regression models.

A two-tailed *P*-value of 0.05 was considered statistically significant. Robust standard errors were estimated and adjusted for clustering at the drug level.

## Results

A total of 276 drug-indication approvals between 2008 and 2022 had corresponding price and efficacy metrics and certainty of evidence data (at least 1 efficacy metric per drug-indication). Most of the approvals concerned lung cancer (19.2%), breast cancer (10.9%), lymphoma (10.1%), and leukemia (7.6%). Of the 276 drug-indication approvals, 65.2% received approval based on an underlying RCT study, 63.8% concerned subsequent indications (as opposed to the original drug approval), and 31% were approved in the accelerated approval pathway. Further, almost two-thirds were approved initially for orphan indication, and only one-quarter showed an improvement in OS (Table S2 in Supplement).

The analysis sample size for relating prices to the efficacy metrics differs for each of the 5 efficacy metrics due to varying data availability, with ESMO-score data available for 166/276 drug-indication approvals with price data, ORR data available for 159/276, PFS data available for 111/276, OS data for 88/276, and QALY data for 51/276.

**Figure 1.**
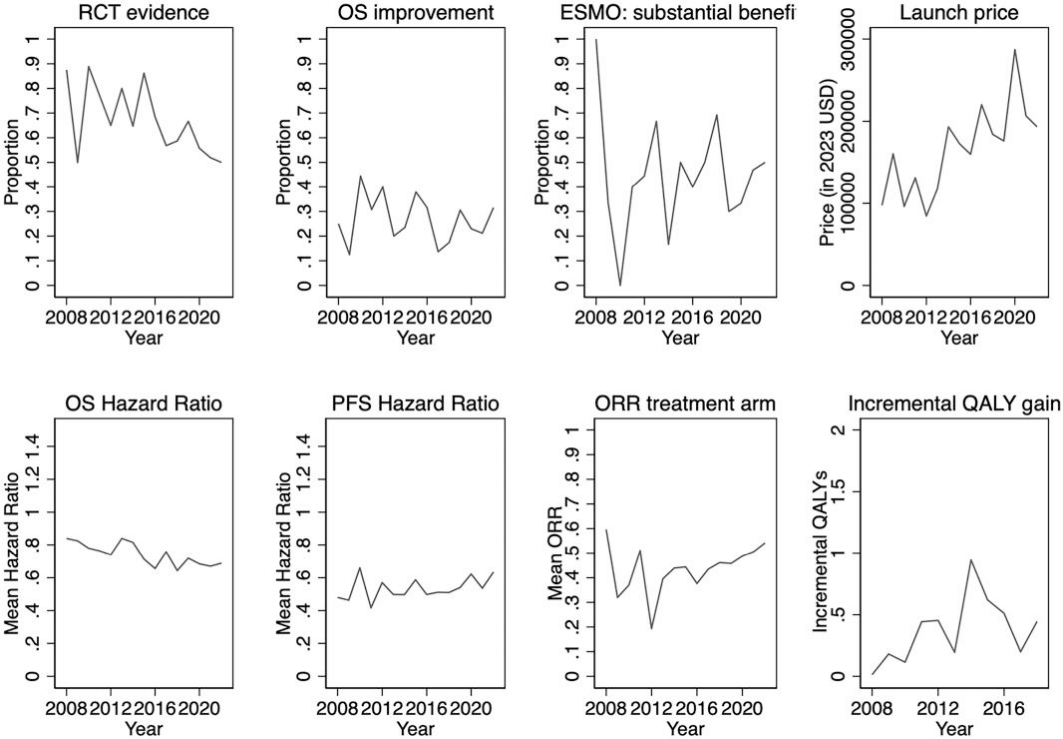
Time trends of launch price, certainty of evidence, and efficacy. ORR, objective response rate; OS, overall survival; PFS, progression free survival; QALY, quality-adjusted life-years; RCT, randomized controlled trial.

Figure 1 shows the time trends for the launch price, efficacy, and certainty of evidence variables. Figure 1 reveals that the proportion of FDA approvals relying on RCT evidence has slightly decreased over time (*P*-value = 0.02), and the average inflation-adjusted launch price has increased from about $100 000 in 2008 to about $200 000 in 2022 (*P*-value = 0.001), or with an average year-to-year increase of about 6%. Among drugs with mature OS data at approval, the mean HR is slightly lower (ie, more beneficial results) in more recent years (*P*-value = 0.001). There is a signal of higher incremental QALY gains in more recent years, but not statistically significant. The time trends for the proportion of approvals based on OS improvements, the proportion with substantial clinical benefit (ESMO scores), mean PFS HRs, and treatment arm ORRs do not reveal any apparent change over time.

**Figure 2.**
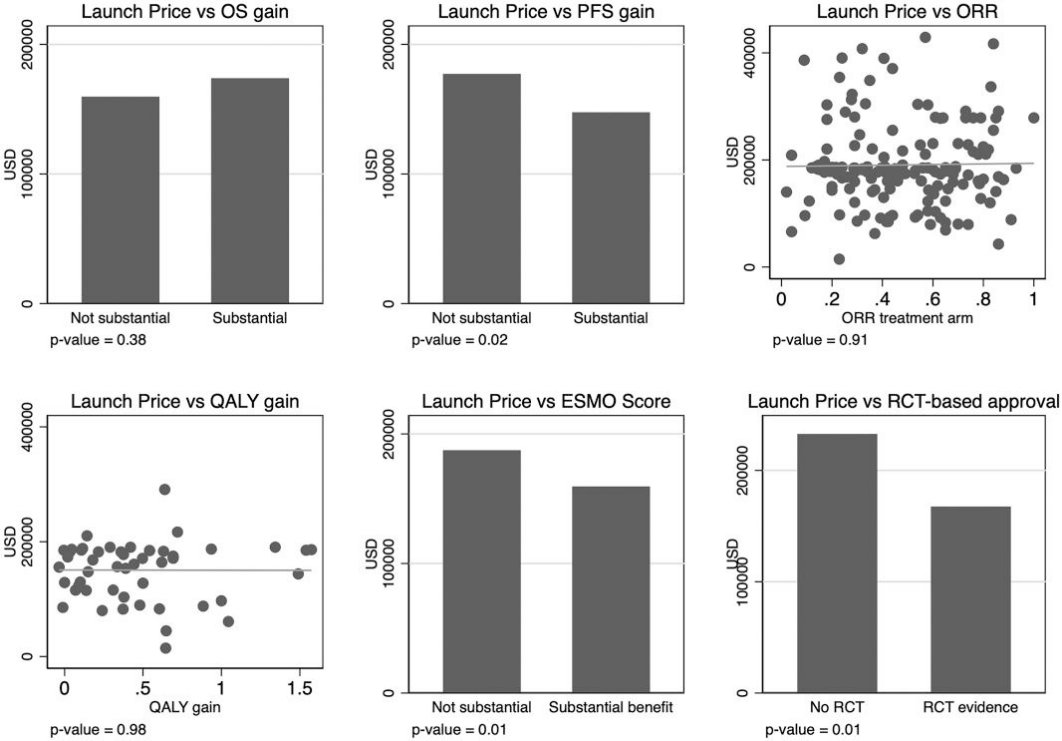
Scatter plots relating launch price to efficacy and certainty of evidence. ORR, objective response rate; OS, overall survival; PFS, progression free survival; QALY, quality-adjusted life-years; RCT, randomized controlled trial.

The raw relationships between the launch prices and the efficacy and certainty of evidence metrics are presented in Figure 2. The launch price is lower for drugs classified by the ESMO score to have substantial clinical benefits and substantial PFS benefits and for drugs where the approval is based on underlying RCTs. No other correlations are statistically significant.

Table 1 shows the regression results, and the only consistent finding in all 5 regression models is that the launch year is statistically significantly associated with higher real launch prices by 5%-8% per year. The coefficient estimates in Table 1 show the association on the logged price, and translating the estimates for the launch year into absolute dollars implies that the yearly inflation-adjusted increase in launch prices (per 1-year of treatment) has been between $7008 and $10 834. Substantial PFS benefits are the only efficacy metrics that show a statistically significant relationship to the launch price, showing a lower launch price for drugs with substantial PFS benefits. It should be noted that the coefficient estimates have wide confidence intervals, indicating considerable uncertainty in the estimates. For example, the 95% CI in the ESMO-score model indicates that a drug with a substantial clinical benefit ranges from a 27% lower to a 1% higher launch price. The RCT evidence variable shows a mixed association with the launch price based on the point estimates, but none of the models indicate statistically significant results. There are some signals that drugs approved in the accelerated approval pathway tend to have higher launch prices, even though the findings are somewhat inconsistent with 3 out of 5 models indicating a statistically and economically significant higher launch price for accelerated approvals.

**Table 1.** Regression results on launch price: coefficient estimates (95% CI).

Variables	Price outcome variable: launch price				
	Model 1	Model 2	Model 3	Model 4	Model 5
OS larger gain	−0.07 (−1.26; 0.90)	—	—	—	—
PFS larger gain	—	−0.18 (−0.34; −0.01)	—	—	—
ORR	—	—	−0.17 (−0.56; 0.21)	—	—
ESMO-score	—	—	—	−0.15 (−0.31; 0.01)	—
QALY-gain	—	—	—	—	−0.15 (−0.38; 0.08)
RCT evidence	−^a^	0.26 (−0.06; 0.59)	−0.08 (−0.25; 0.10)	−0.09 (−0.26; 0.08)	0.24 (−0.13; 0.61)
Orphan disease	0.21 (−0.02; 0.44)	0.10 (−0.10; 0.29)	0.11 (−0.12; 0.34)	0.22 (0.03; 0.41)	0.01 (−0.23; 0.26)
Accelerated approval	0.58 (0.39; 0.76)	0.30 (0.06; 0.53)	0.06 (−0.12; 0.23)	−0.01 (−0.16; 0.31)	0.28 (0.06; 0.50)
Launch year	0.05 (0.02; 0.08)	0.06 (0.03; 0.09)	0.05 (0.02; 0.08)	0.05 (0.03; 0.08)	0.08 (0.02; 0.13)
Observations	88	111	159	166	51
*R* ^2^	0.44	0.34	0.15	0.34	0.22

^a^RCT evidence is perfectly collinear with underlying trials that reported OS hazard rates.

ORR, objective response rate; OS, overall survival; PFS, progression free survival; QALY, quality-adjusted life-years; RCT, randomized controlled trial.

Sensitivity analyses (Online Table S3) were in line with the main results and did not indicate any clear relationship between clinical efficacy and launch prices for the initial vs subsequent indications.

## Discussion

The net (inflation-adjusted) launch price for cancer drugs has increased by about 6% per year over the last 15 years. Using price data accounting for rebates and discounts and measuring clinical efficacy with 5 different metrics, the study results indicated no consistent and statistically significant relationship such that higher-priced drugs have more impressive efficacy outcomes. If anything, some point estimates indicated the opposite, ie, that drugs lacking substantial efficacy improvements were associated with higher net launch prices. These study results are consistent with recent findings showing no consistent relationship between launch “list” prices, not accounting for rebates/discounts, and efficacy metrics.^19,21-25^

Further, the study results did not reveal any consistent relationship between net launch prices and a higher certainty of evidence around the clinical efficacy. The simple bivariate correlations did show a negative correlation between drugs having RCT-evidence for the clinical efficacy and launch prices, but this association disappeared in the regression analyses. The regression analyses did show, however, some indications that prices were higher for drugs approved in the accelerated approval pathway (3 out of 5 analysis samples indicated a statistically significant relationship).

In sum, the findings can be interpreted such that cancer drug prices in the US are not value-based. It has been argued that countries that have chosen alternative prescription drug market regulations using health technology assessments and cost-effectiveness analyses are more likely to see value-based prices.^37^ However, the lack of a relationship between cancer drug prices and clinical efficacy has also been documented in key European markets.^24^ This indicates the lack of value-based prescription cancer drug prices may be a more general global phenomenon.

The lack of evidence for value-based prices in this study should be interpreted with caution considering a number of study limitations. First, while we improved upon previous studies by using net price data that account for rebates and discounts, the analyses are still limited by the availability and accuracy of this data. Although we controlled for time trends and a few additional covariates, there is undoubtedly potential for unmeasured confounding that may hinder us from identifying a possible “true” price-efficacy relationship. Also, the wide confidence intervals in our regression models indicate a high level of uncertainty around the point estimates, allowing for both relatively large negative and positive relationships. Additionally, there is a lot of missing data for the global set of FDA-approved cancer drugs due to substantial missing launch price information and efficacy data. There are some patterns over time in the share of missing data, with more missing data in recent years for OS and PFS data, whereas there is less missing data in the most recent years for the ESMO score and ORR data. The fact that we use several efficacy metrics can reduce the risk that systematic missingness in a single efficacy variable unduly influences the results and interpretations, but there is still limitations and uncertainty around the high share of missing information.

In terms of implications, the potential lack of value-based cancer drug launch prices can imply that the prescription drug market does not provide efficient economic incentives for producers, patients, and society. This has also been discussed in a previous study focusing on pricing for cancer drugs in the accelerated approval pathway, finding that there are no price gains for producers when confirming clinical benefits.^38^ If there are no clear additional financial incentives for developing drugs with more meaningful clinical benefits, this may lead to an inefficient allocation of research and development investments.^39^ In sum, paying producers substantially more for the most effective cancer drugs can lead to a higher supply of such drugs.^40^

## Supplementary Material

qxaf051_Supplementary_Data
