# Supplementary material for: An analysis of US net cancer drug launch prices and clinical efficacy and certainty of evidence from 2008 to 2022
Source: Health Aff Sch. 2025 Mar 13;3(4):qxaf051. doi: 10.1093/haschl/qxaf051 (PMC11970244; doi:10.1093/haschl/qxaf051)
Supplement: qxaf051_Supplementary_Data [file qxaf051_supplementary_data.zip › Supplement (5).docx]

**Online supplement for “The Relationship between US Cancer Drug Launch Prices and Clinical Efficacy and Certainty of Evidence: An Analysis Using Net Price Data from 2008 to 2022”**

**Table S1.** Data extraction template

| **Item** | **Description** |
| --- | --- |
| Brand | Brand name of approved drug |
| Generic | Generic name of approved drug |
| Indication | Type of cancer indication |
| Date of approval | Date |
| Solid tumor | Drug targeting solid or non-solid tumor |
| First indication | First approval for drug (or subsequent approval) |
| Orphan status | If drug is given orphan status for the approved indication |
| Accelerated approval | If drug is approved in the accelerated approval pathway |
| RCT evidence | If pivotal trial(s) were randomized (or single-arm) |
| OS evidence | If pivotal trial(s) reported overall survival gains |
| ESMO score | ESMO-MCBS score 1-5 |
| OS-HR | OS hazard ratio from the pivotal trial(s) |
| OS median | Improvement in median life expectancy from pivotal trial(s) |
| PFS availability | PFS data available from pivotal trial(s) |
| PFS-HR | PFS hazard ratio from the pivotal trial(s) |
| PFS median | Improvement in median PFS |
| ORR availability | ORR data available in the pivotal trial(s) |
| ORR gain | Percentage of patients with ORR from the pivotal trial(s) |
| QALY gain | Incremental QALY gain for the drug compared to standard of care |

**Notes:** RCT = Randomized Controlled Trial, OS = Overall Survival, ESMO-MCBS = ESMO Magnitude of Clinical Benefit Scale, PFS = Progression-Free Survival, ORR = Objective Response Rate.

**Table S2.** Sample summary statistics for FDA cancer drug approvals 2008 – 2022

| **Indication** | **Approvals N (%)** | **Accelerated approval N (%)** | **RTC study design N (%)** | **Verified OS N (%)** |
| --- | --- | --- | --- | --- |
| All | 276 (100) | 86 (31.16) | 180 (65.22) | 74 (26.81) |
| Bile duct/pancreas cancer | 3 (1.09) | 1 (1.16) | 2 (1.11) | 2 (2.70) |
| Urothelial/bladder cancer | 11 (3.99) | 6 (6.98) | 3 (1.67) | 2 (2.70) |
| Bone and Soft Tissue tumors | 4 (1.45) | 1 (1.16) | 2 (1.11) | 2 (2.70) |
| Brain and Nervous System Cancer | 4 (1.45) | 3 (3.49) | 1 (0.56) | 1 (1.35) |
| Breast cancer | 30 (10.87) | 7 (8.14) | 27 (15.00) | 5 (6.76) |
| Colorectal cancer | 8 (2.90) | 1 (1.16) | 7 (3.89) | 6 (8.11) |
| Endocrine tumors | 11 (3.99) | 4 (4.65) | 5 (2.78) | 0 (0.00) |
| Gastric or esophageal cancer | 16 (5.80) | 2 (2.33) | 14 (7.78) | 11 (14.86) |
| Gynecological cancer | 10 (3.62) | 3 (3.49) | 5 (2.78) | 2 (2.70) |
| Head and neck cancer | 4 (1.45) | 1 (1.16) | 3 (1.67) | 3 (4.05) |
| Hepatocellular carcinoma | 5 (1.81) | 2 (2.33) | 3 (1.67) | 2 (2.70) |
| Leukemia (all types) | 21 (7.61) | 9 (10.47) | 13 (7.22) | 3 (4.05) |
| Leukemia (all types) or lymphoma (all types) | 7 (2.54) | 0 (0.00) | 6 (3.33) | 1 (1.35) |
| Lymphoma (all types) | 28 (10.14) | 15 (17.44) | 9 (5.00) | 1 (1.35) |
| Lung cancer | 53 (19.20) | 15 (17.44) | 35 (19.44) | 20 (27.03) |
| Multiple myeloma | 10 (3.62) | 4 (4.65) | 8 (4.44) | 0 (0.00) |
| Prostate cancer | 12 (4.35) | 1 (1.16) | 11 (6.11) | 7 (9.46) |
| Renal cell carcinoma | 12 (4.35) | 0 (0.00) | 12 (6.67) | 3 (4.05) |
| Skin cancer | 20 (7.25) | 5 (5.81) | 14 (7.78) | 3 (4.05) |
| Other | 7 (2.54) | 6 (6.98) | 0 (0.00) | 0 (0.00) |

**Notes:** The analysis sample consists of 276 FDA cancer drug approvals with available price and efficacy data (from at least one of the five efficacy metrics used in the analysis).

**Table S3.** Sensitivity analyses: Coefficient estimates (95% CI)

| Efficacy variable | All indications | | First indication | | Subsequent indications | |
| --- | --- | --- | --- | --- | --- | --- |
|  | OLS | GLM^*^ | OLS | GLM^*^ | OLS | GLM^*^ |
| OS higher gain | -0.05  (-0.25;0.15) | -0.01  (-0.19;0.19) | 0.01  (-0.36;0.38) | 0.05  (-0.27;0.37) | -0.13  (-0.41;0.15) | -0.08  (-0.31;0.16) |
| PFS higher gain | -0.16  (-0.32;-0.01) | -0.15  (-0.31;0.01) | -0.12  (-0.34;0.10) | -0.11  (-0.33;0.11) | -0.19  (-0.40;0.02) | -0.18  (-0.38;0.02) |
| ORR | -0.09  (-0.45;0.28) | -0.14  (-0.54;0.26) | -0.06  (-0.63;0.52) | -0.27  (-0.84;0.30) | -0.14  (-0.47;0.20) | -0.05  (-0.37;0.26) |
| ESMO-score | -0.17  (-0.32;-0.02) | -0.15  (-0.27;-0.03) | -0.34  (-0.69;0.01) | -0.22  (-0.49;0.05) | -0.09  (-0.22;0.05) | -0.08  (-0.20;0.04) |
| QALY-gain | -0.12  (-0.36;0.12) | -0.04  (-0.21;0.13) | -0.21  (-0.58;0.16) | -0.10  (-0.37;0.17) | 0.09  (-0.05;0.22) | 0.08  (-0.01;0.17) |

**Notes:** The result in each cell represents a separate regression model with launch year as the only additional covariate. The OLS models use the logged launch price as the dependent variable (as in Table 1). The GLM models are, based on best statistical fit, assuming a log link and gamma family distribution. Standard errors are robust and clustered at the brand level.

**Note S1.** Note on outcome variable definition

Definition of the launch price variable

The definition of the *Price_Launch_* variable is exemplified in the figure below for a hypothetical drug X that receives FDA approval sometime in Q1 2020. We assign the price to equal the first available price data from any of the two quarters following the FDA approval. There is frequently a gap in the database such that the first available price data comes in in the second quarter following the FDA approval (i.e., Drug X is approved in Q1 2020, and the first price data becomes available in Q3 2020).

For drugs approved for subsequent indications (e.g., Drug X was previously approved for breast cancer, and Q1 2020 now receives approval for lung cancer), price data is typically available for the same quarter as the FDA approval. We still base the launch price on the first available price data in any of the two following quarters since we want to have room to capture any price adjustments that may happen due to the new indication.

2018

2019

Drug X FDA-approval in

Q1 2020

*Price_Launch_* = first available price data in Q2-Q3 2020

2020

2022

2021

2023

2024

Year

**Missing net price data adjustments**

The adjustment for missing net price data is based on assuming a fixed price discount. For a Drug X that is approved in Q1 2020 and where the first net price data becomes available in, for example, Q4 2022, we assume that the discount seen in Q4 2022 also applied for the launch price period (as defined above) and apply this to the gross price (Q2-Q3 2020).
